# The effect of coping strategies on health–related quality of life in acromegaly patients

**DOI:** 10.1007/s12020-024-03813-4

**Published:** 2024-04-13

**Authors:** Lisa Schock, Witold X. Chmielewski, Sonja Siegel, Mario Detomas, Timo Deutschbein, Sabrina Giese, Jürgen Honegger, Nicole Unger, Ilonka Kreitschmann-Andermahr

**Affiliations:** 1 Department of Neurosurgery and Spine Surgery, Member of ENDO-ERN, University Hospital Essen, Essen, Germany; 2 German Cancer Consortium (DKTK) Partner Site, University Hospital Essen, Essen, Germany; 3 Center for Translational Neuro- & Behavioral Sciences (C-TNBS), University Duisburg Essen, Essen, Germany; 4 Department of Internal Medicine I, Division of Endocrinology and Diabetes, University Hospital Würzburg, Würzburg, Germany; 5 Medicover Oldenburg MVZ, Oldenburg, Germany; 6 Department of Neurosurgery, Eberhard-Karls-University Tübingen, Tübingen, Germany; 7 Department of Endocrinology, Diabetes and Metabolism, Member of ENDO-ERN, University Hospital Essen, Essen, Germany

**Keywords:** Acromegaly, HRQoL, Coping strategies, Health variables

## Abstract

**Purpose:**

Patients with acromegaly oftentimes exhibit a reduced physical and psychological health-related quality of life (HRQoL). Maladaptive coping styles are associated with poor HRQoL in a number of diseases and patients with pituitary adenomas in general exhibit less effective coping styles than healthy controls. This study aimed to assess coping strategies in acromegaly patients in order to explore leverage points for the improvement of HRQoL.

**Methods:**

In this cross-sectional study, we administered self-report surveys for coping strategies and HRQoL (Short Form SF-36, Freiburg questionnaire on coping with illness, FKV-LIS) in patients with acromegaly. These were set into relation with a variety of health variables.

**Results:**

About half of the 106 patients (44.3% female) with a mean age of 56.4 ± 1.3 years showed impaired physical and psychological HRQoL on average 11.2 years after the initial diagnosis. Body mass index, age at survey date and concomitant radiotherapy explained 27.8% of the variance of physical HRQoL, while depressive coping added an additional 9.2%. Depressive coping style and trivialization and wishful thinking were pivotal predictors of an impaired psychological HRQoL with a total explained variance of 51.6%, whereas patient health variables did not affect psychological HRQoL.

**Conclusion:**

Our results show that maladaptive coping styles have a substantial negative impact on psychological HRQoL in patients with acromegaly, whereas physical HRQoL is influenced to a lesser extent. Specialized training programs aimed at improving coping strategies could reduce long-term disease burden and increase HRQoL in the affected patients.

## Introduction

Acromegaly is a rare and severe endocrine disorder, which is caused by unrestrained growth hormone (GH) secretion occurring after the closure of the epiphyseal plates at puberty [1, 2]. The prevalence of acromegaly is estimated at between 2.8 and 13.7 in 100,000, based on the examined population, and in 95% of cases acromegaly is caused by a GH-secreting pituitary adenoma [2]. While the clinical features of acromegaly and its comorbidities have been investigated in depth for years [3], psychological aspects and health-related quality of Life (HRQoL) of the disease have been deeply analyzed only recently. The latter research has led to the insight that patients with acromegaly are more prone to develop manifest affective disorders than healthy controls [4–6] and may suffer from a reduced overall HRQoL, even years after initial diagnosis or successful treatment of GH hypersecretion [1, 7]. These data suggest that improving HRQoL in acromegaly is pivotal in order to alleviate the allostatic load associated with the disease, a cumulative effect of both stressful life events and chronic stress [8].

A variety of factors influencing HRQoL in patients with acromegaly has been discussed. It has been shown that type of treatment and patient age have an effect on psychological HRQoL in acromegaly; physical HRQoL was predicted in one study by the delay between first symptoms of the disease and diagnosis, body mass index (BMI), number of doctors visited before the diagnosis of acromegaly, and age at diagnosis [1]. In other chronic diseases, such as obesity and various types of cancer, a similar pattern of unmodifiable or hard to modify HRQoL predictors has been observed [9–14]. On the other hand, psychological and physical HRQoL are also massively affected by the thoughts and behaviors mobilized by patients to manage their illness, a behavior called coping [15]. Numerous studies have shown the influence of coping on HRQoL in patients with various medical conditions, ranging from multiple sclerosis to congestive heart failure and cancer [16–19]. Healthy coping has also been identified as one of the key self-care behaviors in patients with diabetes, a wide-spread endocrine disorder [20].

Preliminary data indicate that coping strategies also influence HRQoL in patients with pituitary adenomas. One study found that patients with functioning and nonfunctioning pituitary adenomas showed different and less effective coping strategies than healthy controls [21] and a further one demonstrated that maladaptive coping styles contribute to psychosocial impairment in patients with Cushing’s disease [22]. In order to extend the knowledge on the effects of coping in pituitary disease, in the present study we investigated the relation of illness coping, HRQoL and a variety of clinical variables in a large cohort of patients with acromegaly. The aim was to define predictors of reduced HRQoL and explore potential leverage points for ameliorating psychological well-being in this severely afflicted patient group.

## Subjects, materials and methods

The study was performed at two tertiary university treatment centers for acromegaly (University of Duisburg-Essen, University of Tübingen) and a large endocrinology practice (Medicover Oldenburg MVZ). It was part of a larger research project on the burden of acromegaly, which aimed to investigate stresses in day-to-day life and needs for assistance of patients with acromegaly. To that end, patients treated for a GH-secreting pituitary adenoma with clinically and biochemically-proven acromegaly in these institutions were asked to fill in a variety of self-report questionnaires, including ones on HRQoL and illness coping, which are the subject of the present analysis. Additional self-developed questionnaires which pertained to the research question of disease burden and support needs were not included into the present analysis and will be reported elsewhere. Clinical data of the participating patients were collected from case records. Exclusion criteria were insufficient fluency of the German language to complete a self-reporting questionnaire and diagnosis of an active psychotic disorder.

### Questionnaires

#### Health-related quality of life (HRQoL)

HRQoL was measured with the German version of the self-report questionnaire Short Form SF-36 [23, 24]. The SF-36 is a cross-disease measure for the evaluation of health treatments in terms of HRQoL. In total, eight z-standardized subdomains, *physical pain, physical functioning, physical role functioning, general and psychological well-being, vitality, social and emotional role functioning* are measured with 36 items. The raw values are transformed for the comparison with the normative population. The standardized (T-values) physical and psychological summary scores have a mean of 50 and standard deviation (SD) of 10 with higher scores indicating a better HRQoL. One SD below mean represents an impaired HRQoL, two SD below mean represents a severely impaired HRQoL.

These summary scores were utilized for the analyses reported in the present paper.

### Coping strategies

The German short version of the Freiburg questionnaire on the use of coping strategies (FKV-LIS) questionnaire was used for the assessment of coping strategies [25]. The FKV-LIS assesses the five different coping strategies *problem-oriented coping, religiousness and search for meaning, distraction and self-affirmation, trivialization and wishful thinking and depressive coping* in regard to dealing with a disease on a cognitive, emotional and behavioral level. The 35 items used are answered on 5-point Likert scales, concerning the agreement with the statement, with the answering options “not at all”, “not much”, “moderate”, “quite”, “very strong”. The scores of different coping strategies are obtained by forming a sum of the corresponding items of each scale and dividing it by the number of items, respectively. Higher scores indicate a stronger expression of the coping strategy.

### Patient health variables

To account for the patients‘ medical history, we included the following health variables: Number of surgeries, concomitant radiotherapy, age at the survey date, duration of illness (since initial diagnosis), sex and body mass index (BMI) in the correlation analyses. In the regression analysis, we then included those health variables, that predicted HRQoL in acromegaly before [1], or correlated significantly with the sum scores of SF-36, or both.

### Statistical analyses

SPSS 29 was used to conduct all statistical analyses. Descriptive values were reported with means and standard error of means (SEM). Valid percent were reported.

Pearson correlation coefficient (r) was used to report correlation. Correlation coefficients in parentheses (|r ≤ |) display absolute values, because plus or minus is irrelevant.

Multiple linear regression analyses were preceded by the verification of the assumptions of linearity, normal distribution of the residues, homoscedasticity, and independence of the residues, as well as absence of extreme outliers and multicollinearity.

Two independent linear regression analyses with the dependent variables physical sum score and psychological sum score of the SF-36 were conducted. As predictors, patient health variables (see 2.4) were included in the first step. Sex was also included in the first step to control for the effects of biological sex. Non-metric variables were dummy coded. In the second step, coping variables that significantly correlated with the sum scores of SF-36 were added to explain additional variance.

In the results section, proportion of explained variance (R^2^) and change in explained variance (ΔR^2^) are reported as well as regression coefficient (β), standard error (SE) and standardized regression coefficient (BETA) from the second step. Significance level was set at *p* ≤ 0.05. We decided against alpha correction in order to decrease the risk of falsely dismissing relevant factors (beta error).

## Results

### Study population

The study population was part of a project on the burden of acromegaly with a total of 127 patients, 32.3% from Essen, 35.5% from Tübingen, 32.3% from Oldenburg. All questionnaires were sent via postal delivery with about one third of returned questionnaires across all centers. For the present study, only data sets without missing values in the regression variables were included, resulting in 106 patients (44.3% female) with a mean age of 56.4 ± 1.3 years and an average of 11.2 ± 0.8 past years after the initial diagnosis. Of these, 95.3% had undergone surgery (77.4% once, 17.0% twice and 0.9% three times) and 17.0% patients had received radiotherapy as part of their treatment. Somatostatin analogs (SSA) were taken by 34.9%, dopamine agonists by 4.7%, and GH-receptor antagonists by 19.8% of the investigated patients; 10.4% were at least double-medicated. In the study sample the following hormone axes were replaced: in 19.1% the gonadotropic axis, in 16.7% the corticotropic axis and in 29.0% the thyrotropic axis. 17.6% of the patients had an abnormal IGF-1 level. In addition, 41.5% of patients were classified as pre-obese (BMI greater than or equal to 25–29.9), 24.5% of patients qualified for obesity grade 1 (BMI 30–34.9), 11.3% qualified for obesity grade 2 (BMI 35–39.9) and 5.7% patients qualified for obesity grade 3 (BMI greater than or equal to 40) [26]. Table 1 gives the clinical details of the included patients.

**Table 1:** Patient characteristics

Variables	Total (*N* = 106)
Sex	
Female	44.3%
Male	55.7%
Age (years)	56.4 ± 1.3
Years after initial diagnosis	11.2 ± 0.8
Radiotherapy	17.0%
Surgery	
Once	77.4%
Twice	17.0%
Three times	0.9%
Hormone replacement therapy	
Thyrotropic axis	29.0%
Gonadotropic axis	19.1%
Corticotropic axis	16.7%
IGF deviated from the norm	17.6%
Medication	
Somatostatin analogs	34.9%
GH-receptor antagonists	19.8%
Dopamine agonists	4.7%
Obesity grade	
Pre-obese	41.5%
Grade 1	24.5%
Grade 2	11.3%
Grade 3	5.7%

Data are displayed as mean ± standard error of the mean or percentage (%) of the whole sample

### SF-36 and Coping strategies

The mean physical summary score of the SF-36 of the patient cohort was 39.3 ± 1.3. In total 48 patients reported impaired (23.6% of patients < −1SD below mean) or severely impaired (21.7% of patients < −2SD below mean) physical HRQoL. On the psychological summary score, patients averaged 44.7 ± 1.1. A total of 47 patients reported impaired or severely impaired psychological HRQoL (17.9% of patients < −1SD and 26.4% of patients < −2SD). Regarding coping strategies according to the FKV-LIS, the patients exhibited a mean score of 2.1 ± 0.1 on *depressive coping*, 2.8 ± 0.1 on *active, problem-oriented coping*, 2.8 ± 0.1 *distraction and self-affirmation*, 2.6 ± 0.1 on *religiousness and search for meaning* and 2.2 ± 0.1 on *trivialization and wishful thinking* (compare Table 2).

**Table 2:** Scores of HRQoL (SF-36) and coping strategies (FKV-LIS)

Variables	Number
SF-36	
Physical summary score	39.3 ± 1.3
impaired physical HRQoL (<−1SD)	23.6%
severely impaired physical HRQoL (<−2SD)	21.7%
Psychological summary score	44.7 ± 1.1
impaired psychological HRQoL (<−1SD)	17.9%
severely impaired psychological HRQoL (<−2SD)	26.4%
FKV-LIS	
depressive coping	2.1 ± 0.1
active coping	2.8 ± 0.1
distraction and self-affirmation	2.8 ± 0.1
religiousness and search for meaning	2.6 ± 0.1
trivialization and wishful thinking	2.2 ± 0.1

Data are displayed as mean ± standard error or percentage (%) of the whole sample

*SD* standard deviation

### Correlation between coping strategies, patient health status and HRQoL

Regarding the effect of patient health variables on the physical summary score of the SF-36, age at the survey date, concomitant radiotherapy and BMI correlated negatively, albeit weakly, with physical HRQoL (all r ≤ −0.200; all *p* ≤ 0.040). Number of surgeries, duration of illness and sex did not correlate significantly (all r ≤ |0.119|; all *p* ≥ 0.226). Moreover, the maladaptive coping strategies *trivialization and wishful thinking*, as well as *depressive coping* also correlated negatively with physical HRQoL (all r ≤ −0.283; all *p* ≤ 0.003). No significant correlations between the physical summary scale and *problem-oriented coping*, *religiousness and search for meaning*, *distraction or self-affirmation* were observed (all r ≤ |0.133 | ; all *p* ≥ 0.174).

In regard of the psychological summary score of the SF-36, no significant correlations with the patient health variables were found (all r ≤ |0.056|; all *p* ≥ 0.567). However, a negative influence of the coping strategies *depressive coping, religiousness and search for meaning* and *trivialization and wishful thinking* was observed (all r ≤ −0.246; all *p* ≤ 0.011). *Active coping* as well as *distraction and self-affirmation* did not correlate significantly with psychological HRQoL (all r ≤ |0.105|; all *p* ≥ 0.285). For detailed results confer to Table 3.

**Table 3:** Correlation between coping strategies (FKV-LIS), patient health status and HRQoL (SF-36)

Variables	SF-36 physical summary score	SF-36 psychological summary score
FKV-LIS		
Depressive coping	−0.321**	−0.665**
Active coping	−0.093	−0.105
Distraction and self-affirmation	−0.100	−0.070
Religiousness and search for meaning	−0.133	−0.246*
Trivialization and wishful thinking	−0.283**	−0.433**
Patient health status		
Number of surgeries	0.003	0.024
Radiotherapy	−0.200*	0.049
Age	−0.305**	0.056
Duration of illness	−0.119	0.042
Sex	−0.083	−0.016
BMI	−0.397**	−0.029

Displayed are Pearson correlation coefficients

*p < 0.05; **p < 0.01, two-tailed

### Predictors of impaired HRQoL

#### Physical HRQoL

Including the physical health variables and sex in the first step of the regression model explained a significant proportion of variance of the physical summary score (27.8%). High BMI, higher age at the survey date and a history of radiotherapy were significant negative predictors of the physical summary score, while sex was not. The addition of coping styles added a significant amount to the explained variance (9.2%), resulting in an explained variance of 37.0%. *Depressive coping* was a significant predictor of reduced physical HRQoL, while *trivialization and wishful thinking* was not.

#### Psychological HRQoL

None of the physical health variables significantly explained the variance of the psychological summary score (0.7%). The inclusion of coping styles in the second step of the regression model added a significant amount to the explained variance (50.9%) to a total explained variance of 51.6%. Regarding coping styles, *depressive coping* and *trivialization and wishful thinking* were significant negative predictors of psychological HRQoL, while *religiousness and search for meaning* was not. Interestingly, sex, which was not a significant predictor in the first step of the regression model, was a significant predictor of psychological HRQoL in the second step.

When conducting the regression analysis for either sex separately, following results were obtained for men and women: For men, neither BMI, age at the survey date, nor concomitant radiotherapy were significant predictors of psychological HRQoL in the first step of the regression analysis (explained variance: 1.5%). The inclusion of coping styles added a significant amount of 53.8% explained variance in the second step of the regression model and led to a total of 55.3% explained variance. For men, only *depressive coping* was a significant contributor to psychological HRQoL. Similarly, for women, physical health variables did not significantly predict psychological HRQoL in the first step of the regression analysis (4.6%). Including coping styles in the second step of the regression analysis increased the explained variance by 47.6% to an explained variance of 52.2%. For women *depressive coping*, *trivialization and wishful thinking* and BMI were significant predictors of psychological HRQoL.

Detailed results of the regression analysis are displayed in Table 4.

**Table 4:** Regression of HRQoL on health variables and coping strategies

Predictor	β^a^	SE^a^	BETA^a^	Sig.^a^	R^2^	ΔR^2^
Predictor of the physical sum score of SF-36						
1 Sex	−4.21	2.26	−0.161	0.066		
Age	−0.24	0.08	−0.250	0.003		
BMI	−0.77	0.20	−0.318	<0.001		
Radiotherapy	−7.35	2.87	−0.213	0.012	0.278**	
2 Depressive Coping	−3.72	1.38	−0.254	0.008		
Trivialization and wishful thinking	−1.45	1.25	−0.109	0.247	0.370**	0.092*
Predictor of the psychological sum score of SF-36						
1 Sex	−4.55	1.81	−0.193	0.014		
Age	0.05	0.06	0.055	0.455		
BMI	0.25	0.16	0.115	0.121		
Radiotherapy	1.02	2.30	0.033	0.657	0.007	
2 Depressive Coping	−9.13	1.18	−0.691	<0.001		
Religiousness and search for meaning	1.13	1.10	0.082	0.306		
Trivialization and wishful thinking	−1.99	0.99	−0.166	0.047	0.516**	0.509**

*β* regression coefficient, *SE* standard error, *BETA* standardized regression coefficient, *Sig*. significance level, *R*^*2*^ proportion of explained variance, *ΔR*^*2*^ change in R^2^

*p = 0.001; **p < 0.001

^a^Coefficients from the final regression model (second step)

## Discussion

Undisputedly, acromegaly is associated with a high disease- and treatment-related burden. All efforts made to minimize the allostatic load of the afflicted patients must therefore be researched [8]. This also includes disease-coping. To our knowledge, this study is the first to predict physical and psychological aspects of HRQoL in a large sample of patients with acromegaly in relation to their coping styles and health-related variables. Moreover, this is the second study which affirms maladaptive coping strategies in acromegalic patients [21]. We found that roughly half of the patients with acromegaly had impaired or severely impaired physical and psychological HRQoL 11.2 years after the initial diagnosis. This finding might indicate that physical and psychological HRQoL progressively worsen in the course of the disease, as only roughly one-third of acromegaly patients exhibited a decreased physical and psychological HRQoL 7.1 years after initial diagnosis in another study by our research group [1].

With BMI, age and concomitant radiotherapy explaining 27.8% of the variance in physical HRQoL in the present sample of acromegaly patients we could confirm the results of a prior study regarding the importance of patient health variables [1]. This findings is well in line with literature showing BMI [9, 10], age [11, 12] and concomitant radiotherapy [13, 14] to impact on HRQoL in patients with other chronic diseases, such as obesity, head and neck and cervical cancer. These results pose a challenge to the treating physician, as - besides obesity (and diabetes), which are rather common features in acromegaly [27, 28] - none of the predictors of impaired physical HRQoL mentioned above can be modified by medical therapy. Regarding coping styles, for physical HRQoL, only depressive coping turned out to be a significant predictor, explaining an additional 9.2% of the variance. A similar effect of a depressive coping style on physical HRQoL has been observed in patients with Cushing’s disease [22], or in a multitude of other non-pituitary medical conditions, as for example inflammatory bowel disease [29], multiple sclerosis [16] or heart failure [17]. This shows that considering health variables and coping styles is important for physical HRQoL in acromegaly [30, 31].

In contrast to physical HRQoL, psychological HRQoL was not predicted by any of the included patient health variables with only 0.7% of explained variance. This finding is not in line with previous results of our group and others showing radiotherapy treatment and age to have an adverse effect on psychological HRQoL in acromegaly [1, 32]. It might, however, be explained by the relatively high age in the present study sample (average age of 56.4).

One of the most relevant results of this study was that maladaptive coping styles as depressive coping and trivialization and wishful thinking were pivotal predictors of poor psychological HRQoL in patients with acromegaly, explaining more than 50% of the variance, especially in the light of the almost non-existent effect (0.7%) of patient health variables. This prediction of HRQoL by means of coping styles extends the findings of Tiemensma and colleagues who already showed the association of maladaptive coping styles with HRQoL in patients with acromegaly and other pituitary pathologies [21]. Similar results have also been confirmed in patients with Cushing’s disease [22], in whom maladaptive coping styles explained one third of the variance of psychological HRQoL.

Our results are also of relevance within the broader socio-psychological concept of illness behavior, referring to any actions or reactions of individuals for the purpose of defining their state of health and obtaining physical or emotional relief from perceived or actual illness [33, 34]. Hence, an improvement of HRQoL - and illness behavior - in patients with pituitary pathology is linked to the use of adaptive coping strategies.

A primary treatment goal in regard of HRQoL should, therefore, be the modification of maladaptive coping strategies, for example in the setting of cognitive behavioral therapy. This could be achieved by means of coping practice, which has already been suggested to be beneficial as part of customized training programs in various diseases [18, 19]. Additionally, patients with acromegaly could also benefit from cognitive behavioral therapy supporting adaptive, problem-oriented and solution-focused coping styles. However, when considering that different maladaptive coping styles predict psychological HRQoL in men (depressive coping) and women (depressive coping, trivialization and wishful thinking), coping practice should be individually adapted for patients with acromegaly based on their individual expression of coping styles. Such specialized training programs could reduce long-term disease burden and increase HRQoL [35]. Moreover, spreading the awareness of the importance of coping styles for HRQoL in patients with acromegaly could guide treating physicians to suggest coping practice or an inquiry for psychotherapy for patients with acromegaly. Besides improving patients’ coping abilities, a reduction of the BMI should also be supported, particularly in women. The results indicate furthermore that the rehabilitation process in acromegaly should encompass early diagnostics of coping strategies, illness behavior and HRQoL.

A strength of the present study is the large sample of acromegaly patients, which allows to draw conclusions based on robust statistical analyses. This enabled us not only to quantify the association of coping styles and HRQoL, but also to show that coping styles predict a fundamental amount of the variance of HRQoL in acromegaly patients. We purposefully decided to also include factors that correlated only weakly significant and to refrain from corrections for alpha errors. The aim was to not falsely exclude factors, which would then not be further investigated.

As a possible limitation, our study participants had a severe course of the disease, which might have led to a response bias; a problem shared with other self-report studies, which cannot be averted. Moreover, we must admit that there may be some circularity in the findings, as in a cross-sectional study design, it is not possible to determine if and how the two constructs of coping styles and HRQoL may influence each other over time. Longitudinal studies are necessary to address the reciprocal effects of coping styles and HRQoL in the course of the disease.

Summing up, our results show that roughly half of the patients with acromegaly suffer from impaired physical and psychological HRQoL. Moreover, we showed that maladaptive coping strategies, i.e. depressive coping and trivialization and wishful thinking, are pivotal predictors of psychological HRQoL and also have, albeit less pronounced, effects on physical HRQoL in patients with acromegaly. In regard of these findings, as well as the observed gender-differences and the less pronounced effect of patient health variables on psychological HRQoL, we recommend the development and validation of specialized gender-specific coping training programs. Such programs should support the implementation of more suitable and less maladaptive coping styles in order to help patients with acromegaly reduce the disease burden. Already now, physicians should be alerted on the huge impact of coping styles as a modifying factor of reducing disease burden in acromegaly.
